# Early detection of nasopharyngeal carcinoma through machine‐learning‐driven prediction model in a population‐based healthcare record database

**DOI:** 10.1002/cam4.7144

**Published:** 2024-03-28

**Authors:** Jeng‐Wen Chen, Shih‐Tsang Lin, Yi‐Chun Lin, Bo‐Sian Wang, Yu‐Ning Chien, Hung‐Yi Chiou

**Affiliations:** ^1^ Department of Otolaryngology–Head and Neck Surgery, Cardinal Tien Hospital and School of Medicine Fu Jen Catholic University New Taipei City Taiwan; ^2^ Department of Medical Education and Research Cardinal Tien Hospital New Taipei City Taiwan; ^3^ Department of Otolaryngology–Head and Neck Surgery National Taiwan University Hospital Taipei Taiwan; ^4^ Department of Education and Research Cardinal Tien Junior College of Healthcare and Management New Taipei City Taiwan; ^5^ School of Public Health Taipei Medical University Taipei Taiwan; ^6^ Institute of Population Health Sciences, National Health Research Institutes Miaoli Taiwan; ^7^ Department of Health and Welfare University of Taipei Taiwan

**Keywords:** head and neck cancer, machine learning, nasopharyngeal carcinoma, prediagnostic

## Abstract

**Objective:**

Early diagnosis and treatment of nasopharyngeal carcinoma (NPC) are vital for a better prognosis. Still, because of obscure anatomical sites and insidious symptoms, nearly 80% of patients with NPC are diagnosed at a late stage. This study aimed to validate a machine learning (ML) model utilizing symptom‐related diagnoses and procedures in medical records to predict nasopharyngeal carcinoma (NPC) occurrence and reduce the prediagnostic period.

**Materials and Methods:**

Data from a population‐based health insurance database (2001–2008) were analyzed, comparing adults with and without newly diagnosed NPC. Medical records from 90 to 360 days before diagnosis were examined. Five ML algorithms (Light Gradient Boosting Machine [LGB], eXtreme Gradient Boosting [XGB], Multivariate Adaptive Regression Splines [MARS], Random Forest [RF], and Logistics Regression [LG]) were evaluated for optimal early NPC detection. We further use a real‐world data of 1 million individuals randomly selected for testing the final model. Model performance was assessed using AUROC. Shapley values identified significant contributing variables.

**Results:**

LGB showed maximum predictive power using 14 features and 90 days before diagnosis. The LGB models achieved AUROC, specificity, and sensitivity were 0.83, 0.81, and 0.64 for the test dataset, respectively. The LGB‐driven NPC predictive tool effectively differentiated patients into high‐risk and low‐risk groups (hazard ratio: 5.85; 95% CI: 4.75–7.21). The model‐layering effect is valid.

**Conclusions:**

ML approaches using electronic medical records accurately predicted NPC occurrence. The risk prediction model serves as a low‐cost digital screening tool, offering rapid medical decision support to shorten prediagnostic periods. Timely referral is crucial for high‐risk patients identified by the model.

## Graphical abstract

The LGB‐driven NPC predictive model showed optimal predictive power (AUROC: 0.83), and effectively differentiated patients into high‐risk and low‐risk groups (HR: 5.85). It serve as a low‐cost digital screening tool, offering rapid medical decision support to shorten prediagnostic periods. Timely referral is crucial for high‐risk patients identified by the model.

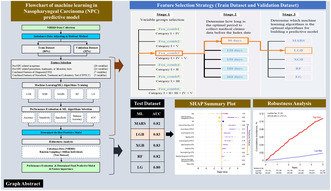

## 1 INTRODUCTION

Nasopharyngeal carcinoma (NPC) arises from the epithelial lining of the nasopharynx,^1^, ^2^ frequently in the pharyngeal recess (Rosenmüller's fossa).^3^ Despite sharing similar cell types with other head and neck cancers, NPC exhibits different risk factors and geographical distribution.^4^, ^5^ NPC is relatively uncommon.^6^ According to the International Agency for Research on Cancer, approximately 133,354 new cases of NPC were identified in 2020, comprising only 0.7% of all malignancies diagnosed that year.^7^ However, in endemic areas, particularly southern China and Southeast Asia,^1^ the prevalence rate and health‐care burden remain high.^8^

Early diagnosis and treatment of NPC are vital for better prognosis,^9^, ^10^ but because of obscure anatomical sites and insidious symptoms, nearly 80% of patients with NPC are diagnosed at a late stage.^11^, ^12^, ^13^ Patients with NPC have relatively long intervals between the first appearance of symptoms and the final diagnosis (the prediagnostic period).^11^ Lee et al.^12^ reported a mean prediagnostic period of 8 months in patients with NPC, with some patients presenting more than 36 months after the first symptom. Patients with late presentations tended to have advanced presenting stages and had significantly poorer disease‐specific survival than those presenting earlier.^10^, ^12^, ^14^

Accredited population‐based screening tools in NPC‐endemic regions remain lacking.^15^ Given the close association between NPC and Epstein–Barr virus (EBV) infection, anti‐EBV IgA serological tests, including VCA‐IgA and EBNA1‐IgA, have been recommended for NPC screening.^16^ However, these tests have a positive predictive value (PPV) as low as approximately 4%,^16^, ^17^ causing >95% of the testing population to undergo unnecessary clinical examinations. Consequently, both compliance and screening efficiency for early diagnosis of NPC remain low. Measurement of circulating plasma cell‐free EBV DNA levels was proposed as a potential screening tool,^1^ but it was discovered to have low sensitivity in identifying patients with early‐stage NPC.^18^ In endemic areas, prevalent latent EBV infection in the general population also caused a high false‐positive rate.^19^, ^20^ These drawbacks have limited the use of EBV DNA as a mass screening tool.

We hypothesized that electronic medical records (EMRs) of symptom‐related diagnoses and reimbursement information in a population‐based database could help detect NPC. However, symptom‐related diagnoses and procedures tend to cluster in groups,^21^ and a complex interplay between them may be challenging to understand. Fortunately, these difficulties can be overcome by using machine learning (ML) approaches.^22^, ^23^, ^24^ Thus, in this study, we inputted demographic data, symptom‐related diagnoses, and procedure reimbursement into an ML algorithm‐based prediction model to evaluate whether such a model could expedite the risk‐stratifying process and shorten the prediagnostic period in patients with NPC.

## 2 METHODS

### 2.1 Study design and participants

We obtained data from Taiwan's National Health Insurance Research Databases (NHIRD)^25^, ^26^ during 2001–2008. The NHIRD is a population‐based medical claims database that includes patient diagnostic, procedural, and treatment information and laboratory testing results. This study was approved by Taiwan's Ministry of Health and Welfare and the Institutional Review Board of Cardinal Tien Hospital (CTH‐110‐3‐5‐013), and the requirement for informed consent was waived because the data in the NHIRD are unidentified.

The NPC diagnosis was identified using the *International Classification of Diseases, Ninth* [*Tenth*] *Revision, Clinical Modification* (*ICD‐9*[*10*]*‐CM*) code 147 and *ICD‐10‐CM* code C11 during the study period. Patients who had cancer before the diagnosis of NPC were excluded. We defined the index date as 14 days before the first diagnosis of NPC to exclude those diagnoses and procedures occurring immediately before confirmation of NPC.

### 2.2 Definition of variables

All claim data within 90, 120, 150, 180, and 360 days before the index date was collected for analysis. Predictor variables were grouped into the following categories (Table S1): (I) participant demographics including sex and age (2 variables); (II) the potential *ICD‐9‐CM* diagnostic codes of pre‐NPC symptoms (28 variables); (III) the potential medical claim data of pre‐NPC procedures, treatments, and laboratory tests (24 variables); (IV) the combined features of diagnostic codes (CFD) (7 variables); and (V) the combined features of procedures, treatments, and laboratory tests (CFPTLT) (5 variables).

### 2.3 Statistical analysis

#### 2.3.1 Model development

**FIGURE 1:**
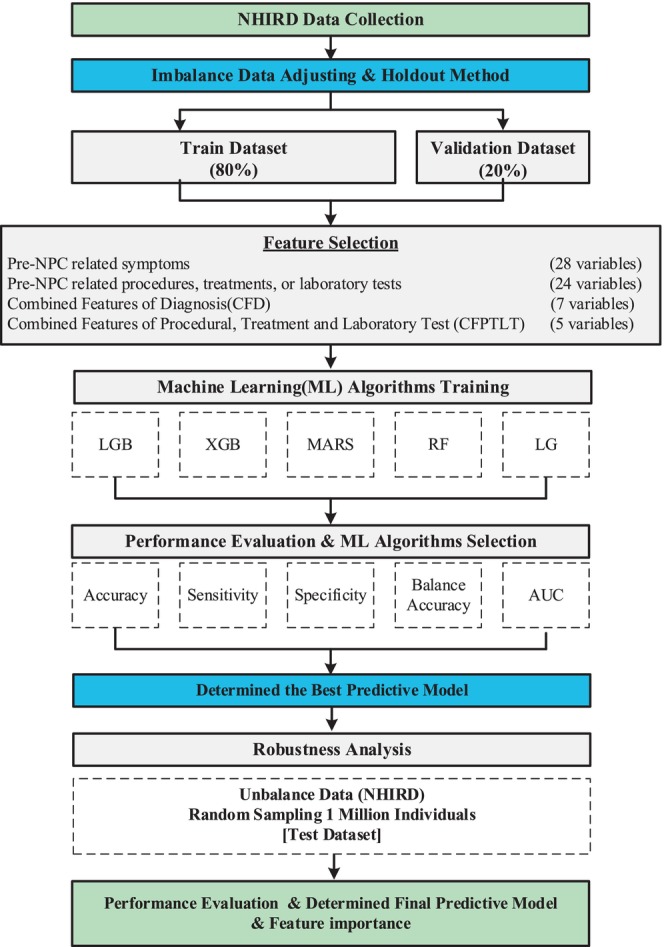
Flowchart of machine learning in Nasopharyngeal Carcinoma (NPC) predictive model. LG, Logistics Regression; LGB, Light Gradient Boosting Machine; MARS, Multivariate Adaptive Regression Splines; NHIRD, the National Health Insurance Research Databases; RF, Random Forest; XGB, eXtreme Gradient Boosting.

Figure 1 depicts the study flowchart. In Taiwan, NPC has an incidence rate of approximately 6.8 per 100,000 population, highlighting a notable contrast between NPC and non‐NPC cases. To mitigate prediction bias, we employed undersampling, achieving a 1:1 ratio between NPC and non‐NPC cohorts by randomly selecting samples from the latter. The data were then divided into training (80%) and validation (20%) sets using the holdout method. Feature selection was performed, and various ML algorithms were compared using the training set, including LGB, XGB, RF, MARS, and LG models. Evaluation metrics included sensitivity, specificity, balanced accuracy, and AUROC. The top‐performing model was applied to the validation set for further assessment. Shapley values were utilized to interpret the model with the highest AUROC.^27^, ^28^ Additionally, to simulate real‐world conditions, we created an imbalanced dataset by sampling 1 million individuals from the NHIRD and assessed the stability of the best model. Finally, the best predictive variables and algorithms were progressively selected to establish the final predictive model.

#### 2.3.2 Feature selection strategy

We employed a two‐step approach for feature selection. First, to mitigate the risk of model overfitting resulting from the inclusion of multiple variables, six sets of feature selection combinations were constructed, derived from categories I–V of Table S1. Table S2 in the Supplement provides detailed information of each feature selection combination used to train the ML algorithms. Subsequently, the models' predictive performance was assessed sequentially to determine the optimal set of feature variables to be included in the final model.

Next, feature combinations from 90, 120, 150, 180, and 360 days before the index date were collected to determine the optimal data length required to achieve the optimal predictive performance. Thus, this study also incorporated the time interval of symptom‐related diagnoses and claims data required for model development.

Data preparation and variable construction were conducted using SAS version 9.4 (SAS Institute). Subsequent ML analyses were performed using R version 3.2.3 (R Foundation for Statistical Computing).

## 3 RESULTS

### 3.1 Baseline characteristics

Table 1 presents the distribution of characteristics between the training and validation sets. A total of 22,186 patients' medical records were included in the analysis. Both sets were balanced for key characteristics except for the “removal of nasal packing” procedure. Eventually, 8874/17748 (50%) patients in the training set and 2219/4438 (50%) in the validation set received 14‐day time window before NPC diagnosis. The 50% proportion of patients with NPC in the training and validation sets resulted from adjustment for imbalanced data. We randomly matched an equivalent number of patients without NPC to achieve balance.

**TABLE 1:** Characteristics of the training and validation sets.

Characteristic	Cohort, No. (%)		*p* Value
	Train data set (*n* = 17,748)	Validation data set (*n* = 4438)	
Age, mean (SD), y	42.62 (18.28)	42.88 (18.50)	0.400
Sex			
Male	11,063 (62.33%)	2754 (62.05%)	0.732
Female	6685 (37.67%)	1684 (37.95%)	
Potential pre‐NPC symptom‐related diagnostic codes			
Benign neoplasm of other and unspecified sites	291 (1.64%)	70 (1.58%)	0.769
Neoplasm of uncertain behavior of other specified sites	5 (0.03%)	0 (0%)	0.263
Neoplasm of uncertain behavior, site unspecified	13 (0.07%)	1 (0.02%)	0.229
Neoplasm of unspecified nature of other specified sites	92 (0.52%)	13 (0.29%)	0.050
Neoplasm of unspecified nature, site unspecified	27 (0.15%)	3 (0.07%)	0.171
Other specified disorders of nervous system	10 (0.06%)	2 (0.05%)	0.773
Unspecified disorders of nervous system	43 (0.24%)	12 (0.27%)	0.736
Trigeminal nerve disorders	93 (0.52%)	21 (0.47%)	0.672
Disorders of other cranial nerves	34 (0.19%)	7 (0.16%)	0.639
Diplopia	28 (0.16%)	4 (0.09%)	0.288
Other disorders of binocular vision	1 (0.01%)	0 (0%)	0.617
Chronic serous otitis media	226 (1.27%)	62 (1.4%)	0.515
Suppurative and unspecified otitis media	591 (3.33%)	141 (3.18%)	0.610
Mastoiditis and related conditions	3 (0.02%)	0 (0%)	0.386
Other disorders of ear	545 (3.07%)	137 (3.09%)	0.955
Hearing loss	306 (1.72%)	71 (1.6%)	0.567
Chronic pharyngitis and nasopharyngitis	786 (4.43%)	216 (4.87%)	0.208
Chronic sinusitis	526 (2.96%)	135 (3.04%)	0.784
Chronic disease of tonsils and adenoids	79 (0.45%)	16 (0.36%)	0.440
Other diseases of upper respiratory tract	269 (1.52%)	71 (1.6%)	0.683
Swelling, mass, or lump in head and neck	930 (5.24%)	214 (4.82%)	0.260
Epistaxis	654 (3.68%)	178 (4.01%)	0.307
Hemorrhage from throat	5 (0.03%)	0 (0%)	0.263
Other symptoms involving head and neck	5 (0.03%)	2 (0.05%)	0.571
Hemoptysis	142 (0.8%)	43 (0.97%)	0.269
Benign neoplasm of connective and other soft tissue of head, face, and neck	192 (1.08%)	52 (1.17%)	0.608
Paralytic strabismus	83 (0.47%)	19 (0.43%)	0.728
Headache	1171 (6.6%)	283 (6.38%)	0.594
Potential NHI claim codes of pre‐NPC symptom‐related procedures, treatments, or laboratory tests			
EBV VCA IgG, IgM, IgA, IFA method, each	840 (4.73%)	211 (4.75%)	0.952
EBV capsid Ab	3 (0.02%)	3 (0.07%)	0.066
EBNA Ab	185 (1.04%)	40 (0.9%)	0.402
Impedance audiometry	228 (1.28%)	63 (1.42%)	0.480
Tympanometry	568 (3.2%)	152 (3.42%)	0.450
Eustachian tube function test	6 (0.03%)	1 (0.02%)	0.705
Nasopharyngolaryngoscopy	2145 (12.09%)	511 (11.51%)	0.294
Sinoscopy	352 (1.98%)	87 (1.96%)	0.922
Laryngoscopy	129 (0.73%)	30 (0.68%)	0.719
Tympanic aspiration	146 (0.82%)	36 (0.81%)	0.940
Myringeal puncture, unilateral	12 (0.07%)	2 (0.05%)	0.593
Middle ear cavity puncture	7 (0.04%)	1 (0.02%)	0.596
Eustachian tube Inflation, unilateral	56 (0.32%)	17 (0.38%)	0.482
Eustachian tube Inflation, bilateral	23 (0.13%)	5 (0.11%)	0.776
Simple epistaxis, anterior	243 (1.37%)	70 (1.58%)	0.293
Complicated epistaxis, posterior	27 (0.15%)	7 (0.16%)	0.932
Intranasal cauterization	1 (0.01%)	0 (0%)	0.617
Anterior nasal packing	14 (0.08%)	6 (0.14%)	0.264
Posterior nasal packing	4 (0.02%)	1 (0.02%)	1.000
Removal of nasal packing	52 (0.29%)	22 (0.5%)	0.036
Tympanocentesis	62 (0.35%)	20 (0.45%)	0.320
Myringotomy UNDER microscope or telescope	91 (0.51%)	13 (0.29%)	0.055
Myringotomy with ventilation tube Insertion under microscope	55 (0.31%)	11 (0.25%)	0.497
Head and neck soft tissue echo	101 (0.57%)	17 (0.38%)	0.128

### 3.2 Feature selection

Figure S1 illustrates the heatmap of the predicted performance (AUROC) of different feature selection combinations and ML algorithms using data from 90, 120, 150, 180, and 360 days before the index date. The vertical axis represents the different types of algorithms used, and the horizontal axis represents the various combinations of feature selection. Regardless of the period of medical records used to establish the predictive model, Fea_comb3 and Fea_comb6 consistently exhibited superior predictive power (Figure S1). However, given the practical and clinical data construction costs, achieving the same level of predictive power with fewer variables is a more compelling solution. Therefore, we selected Fea_comb3 (consisting of 14 variables) for modeling.

We further analyzed the modeling performance of Fea_comb3 variable combinations in various algorithms (Table S4). Regardless of the duration of medical records (90–360 days), the average predictive power of the model remained stable at 0.89, indicating a limited contribution of more days to the model's predictive power beyond 90 days. On the basis of these findings, our model was constructed using the Fea_comb3 variable combination and medical records from 90 days before the index date as the basis for data analysis in modeling.

### 3.3 Predictive ABILITY and validation of performance

Table 2 presents the performance metrics of the ML algorithms based on the selected feature of the Fea_comb3 model collected 90 days before the index date. All models performed well on the test set. LGB and XGB had the same AUROCs, which were slightly higher than those of MARS, RF, and LG. LGB also had slightly higher specificity than XGB.

**TABLE 2:** Performance metrics of machine learning algorithms in 90 days.

	Days before the index date			
	Sensitivity	Specificity	Balanced accuracy	AUROC
A. AUROC value in the training set				
MARS	0.79	0.79	0.79	0.88
LGB	0.81	0.81	0.81	0.90
XGB	0.81	0.80	0.80	0.90
RF	0.79	0.80	0.80	0.90
LG	0.82	0.75	0.78	0.88
B. AUROC value in the validation set				
MARS	0.79	0.79	0.79	0.88
LGB	0.79	0.80	0.79	0.89
XGB	0.80	0.80	0.80	0.90
RF	0.79	0.80	0.79	0.89
LG	0.81	0.75	0.78	0.87
C. AUROC value in the test set				
MARS	0.63	0.80	0.72	0.82
**LGB**	**0.64**	**0.81**	**0.72**	**0.83**
XGB	0.64	0.80	0.72	**0.83**
RF	0.64	0.81	0.72	0.82
LG	0.66	0.76	0.71	0.80

*Note*: This performance metric is based on the selected features of the Fea_comb3 model collected 90 days before the index date.

Abbreviations: LG: Logistics Regression; LGB: Light Gradient Boosting Machine; MARS: Multivariate Adaptive Regression Splines; RF: Random Forest; XGB: eXtreme Gradient Boosting.

Our LGB model achieved an AUROC level of 0.83 using only 14 predictive variables, which was superior to that of the predictive model incorporating all 66 variables (Table S5) in terms of sensitivity, specificity, and AUROC. Consequently, we selected the LGB‐driven model as the final predictive model.

### 3.4 SHAP summary plot

**FIGURE 2:**
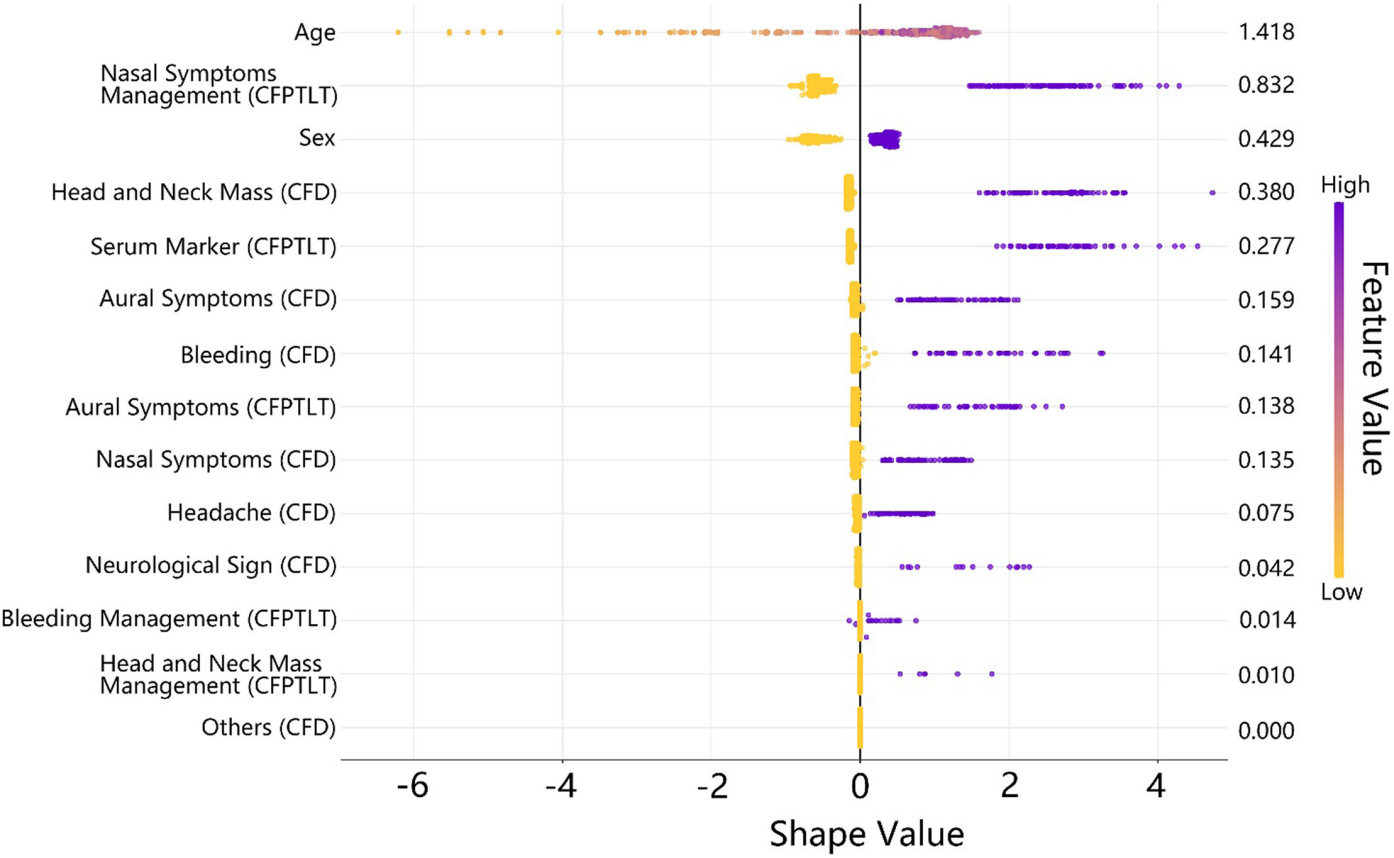
SHAP value of the top 14 features of LGB algorithms. CFD, Combined Features of Diagnostic Codes; FCPTLT, Combined Features of Procedures, Treatments, and Laboratory Tests. This predictive model is based on the selected features of the Fea_comb3 model collected 90 days before the index date.

Figure 2 displays the SHapley Additive exPlanations (SHAP) summary plot,^27^, ^28^ based on the selected features of the Fea_comb3 model collected 90 days before the index date. It demonstrates the ranking of importance and directional influence of the feature variables. The 10 most important variables in descending order were age, nasal symptoms management (CFPTLT), sex, head and neck mass (CFD), serum markers (CFPTLT), aural symptoms (CFD), bleeding (CFD), aural symptom‐related treatments (CFPTLT), nasal symptom‐related treatments (CFD), and headache (CFD). These variables were positively correlated with NPC, indicating a higher likelihood of individuals exhibiting these features within the past 90 days to be diagnosed as having NPC.

### 3.5 Robustness analysis

To further validate the accuracy of the final model in predicting NPC by using real‐world data, we tested it on the data of 1 million individuals randomly selected from the NHIRD as of January 1, 2009. The high‐risk and low‐risk groups were distinguished using the 75th percentile of the risk prediction value (descriptive statistics of different risks are shown in Table S6). The Kaplan–Meier method was used to determine whether individuals predicted to be at high risk by the model had a higher cumulative incidence of NPC in the subsequent 5 years.

**FIGURE 3:**
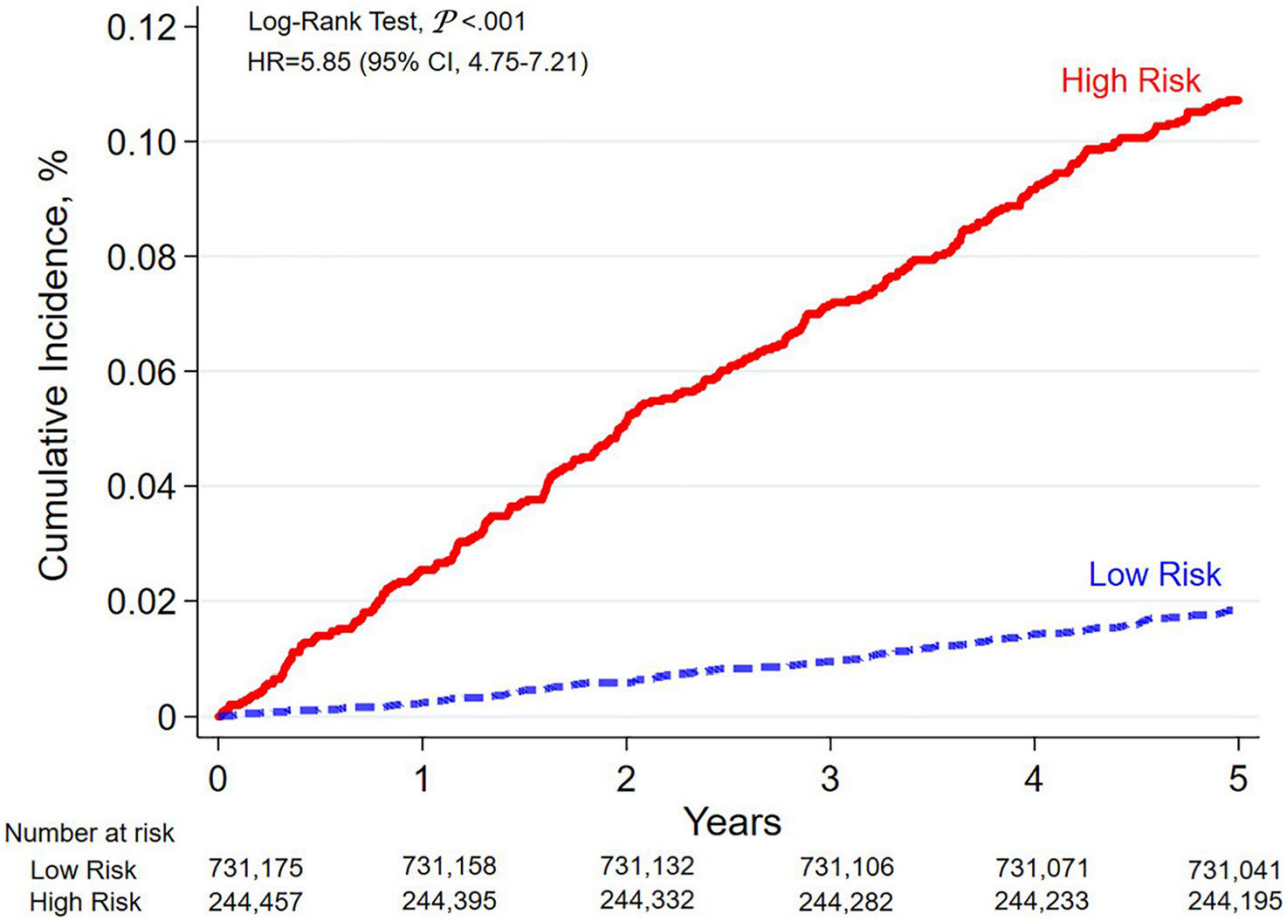
Actual NPC 5‐year incidence under risk prediction based on LGBM algorithms. The study utilized the final prediction model to predict the risk of NPC in a one‐million individuals test dataset randomly selected from the NHIRD database as of January 1, 2009.The high‐risk and low‐risk groups were distinguished using the 75th percentile of the risk prediction value. The incidence rate for the high‐risk group was 21.45 per 100,000, whereas that for the low‐risk group was 3.67 per 100,000, which was approximately 5.85 times (95% CI, 4.75–7.21) lower.

Figure 3 displays the actual NPC 5‐year incidence under risk prediction based on LGBM algorithms. The incidence rate for the high‐risk group was 21.45 per 100,000, whereas that for the low‐risk group was 3.67 per 100,000, which was approximately 5.85 times (95% CI, 4.75–7.21) lower. The model‐layering effect is valid.

## 4 DISCUSSION

To our knowledge, this population‐based cohort study is the first to develop and validate an ML‐based NPC prediction model by using a population‐base medical claims database in an Asian population. With minimal curation of the collected data, our model builds on an otolaryngologist's knowledge in terms of feature selection, including demographic characteristics and NPC symptom‐related diagnoses, procedures, treatments, and laboratory tests. By using routinely obtained information available in a population‐based claims database and only 14 variables, our algorithm exhibited good accuracy in predicting NPC occurrence in the general population. The result of Shapley's summary plot confirmed the explanatory power of our model. Application of this risk‐stratifying tool to personal health‐care records, such as My Health Bank,^29^, ^30^ can help shorten the NPC prediagnostic period. Moreover, this theoretical approach can be scaled across specialties or tailored to predict other critical illnesses by using disease‐specific feature combinations, particularly those with inconspicuous initial symptoms.

The NPC is multifactorial. Although its exact cause remains unknown, three major risk factors have been identified: genetic susceptibility, environmental factors, and EBV infection.^31^ Individuals with a family history of NPC have an increased NPC risk.^32^ This genetic susceptibility is further highlighted by the higher prevalence of NPC in distinctive ethnic groups and geographic regions, particularly in Chinese, Southeast Asian, and North African ethnicities.^33^ Ingesting preserved or salted fish, especially during childhood, has been associated with an increased risk of NPC in populations in which NPC is endemic.^33^, ^34^ Further, dietary exposure to volatile nitrosamines^35^ and occupational exposure to formaldehyde, wood dust, fumes, and chemicals have been identified as environmental risk factors for NPC because these substances produce active carcinogenic metabolites that cause chronic inflammation in the nasopharynx.^36^ Finally, despite the high correlation between EBV infection and NPC, how EBV infection contributes to NPC pathogenesis remains unclear and may be a result of a complex interaction between the host stroma and EBV as well as genetic changes in infected host cells.^37^ Although these predisposing factors contribute to NPC development, practical primary preventive efforts have been limited by the insufficient explanatory power of modifiable risk factors.^38^ Therefore, secondary prevention using screening to detect early and asymptomatic disease has been emphasized. Unfortunately, no widely accepted risk assessment system is available for early‐stage NPC identification, particularly for mass screening in an endemic area.

Several risk assessment models have been developed for the early detection of NPC. In a large case–control study of Cantonese‐origin participants, Ruan et al.^39^ demonstrated that an environmental model that included the factors of salted fish, preserved vegetable consumption, and cigarette smoking could discriminate participants with NPC from those without NPC with only modest ability (AUROC = 0.68). Adding data on the family history of NPC and genetic risk score increased the model performance to AUROCs of 0.70 and 0.74, respectively. However, they did not incorporate the presence of EBV antibody titers, and the model was not validated on an independent data set. Subsequent studies have attempted to combine EBV subtypes, host genetic susceptibility, and serological EBV titers for NPC risk stratification.^40^, ^41^ Zhou et al.^40^ developed a comprehensive NPC risk score incorporating epidemiology factors, 2 host single nucleotide polymorphisms (SNPs), and 3 EBV SNPs, which yielded an AUROC of 0.77 in distinguishing patients with and without NPC in the validation set. Their model improved the PPV for detecting NPC from 4.7% for serum EBV antibody levels alone to 43.24% by including serological test results with the top 20% comprehensive risk score, but at the expense of a decreased negative predictive value from 99.97% to 99.91%. Similarly, He et al.^41^ created and validated a polygenic risk score for NPC derived from a genome‐wide association analysis. The PPV of the model increased from an average of 4.84% to 11.91% when the top 5% of the polygenic risk score and the findings of the EBV‐serology‐based screening test were combined. Nevertheless, the universal applicability of these models in the general population and their cost‐effectiveness remain undetermined, necessitating the development of more practical and cost‐effective models independent of EBV and other laboratory tests for rapid NPC screening of the general population.

Numerous studies have addressed ML applications in health care,^22^, ^23^, ^24^ opening new possibilities for better clinical decision support.^42^, ^43^, ^44^, ^45^, ^46^, ^47^ Chen et al.^48^ designed an algorithm‐driven risk prediction model for NPC screening by using a single institution's EMRs and patient graph analysis, without incorporating EBV and other laboratory test results, to improve accessibility and increase NPC screening rate. The XGB models based on 100 and 20 variables achieved AUROCs of 0.934 and 0.854, respectively, in NPC prediction. However, data collection and preprocessing during model development were time‐consuming. Furthermore, the proposed model consisted of only hospital EMRs but not outpatient clinic records, which may decrease its performance in rural areas. In addition, the model was not tested on an independent data set. By contrast, we used a population‐based claims data set for model development, which included hospital‐based, emergency‐department‐based, and outpatient‐clinic‐based information. Moreover, the feature selection of the model was based on symptom‐related diagnoses, procedures, treatments, and laboratory tests, which were all explainable. We tested our model on an independent data set (2009–2013), achieving high performance. Using the online portal My Health Bank, which contains the individual's medical care data over the past 3 years, Taiwan's citizens with National Health Insurance can check their medical records at any time and monitor their health.^29^, ^30^ This approach provides the potential for personalized risk stratification and large‐scale population screening in endemic areas for the early diagnosis and secondary prevention of NPC.

### 4.1 Strengths

This study has several strengths. First, while the treatment prognosis of NPC has improved in the past decade in Taiwan, patients with NPC who presented with clinical stage III and stage IV increased from 70.4% (961/1365) in 2009 to 72.1% (998/1385) in 2021, indicating a need for a universal screening method to expedite early diagnosis. This study proposed a machine‐learning model using the healthcare‐seeking records available for individual patients (My Health Bank) and nationwide (the National Health Insurance Research Database). Applying the model in screening may alter the presenting stage of patients with NPC in the long run, thereby further improving prognosis. Second, our results indicate that ML can be a promising method for NPC risk stratification. The developed model used a 14‐day time window before NPC diagnosis, a minimum of 90 days of EMR data, and 14 clinically explainable variables. Third, this tool was tested on an independent data set, achieving high performance. By using accessible, personalized data, namely that from My Health Bank, our model could perform an automated NPC risk assessment within seconds, thus facilitating the large‐scale, nationwide implementation of screening programs for early detection of NPC. The decision support system may also assist a physician's clinical decision‐making for NPC diagnostic interventions, especially for nonotolaryngologists. Finally, this approach can be scaled across specialties or customized for risk stratification of other severe diseases, especially those with subtle initial symptoms.

### 4.2 Limitations

This study has several limitations. First, the case–control study‐based predictive model included only sex, age, and symptom‐related management data but no other identified risk factors. A well‐designed cohort study including more established risk factors, such as family history, cigarette smoking, diet, environmental exposure, EBV status, and genetic predisposition, might improve the current model. Second, our model was built using data from a Taiwanese population in an endemic region, precluding easy generalizability to other areas without NPC endemicity and with different ethnicities. Moreover, differences in medical insurance systems and health‐seeking behavior in different countries may cause variations in health‐care data structure and availability. Therefore, the models should be adjusted before they can be used for NPC screening and risk assessment in other countries. Finally, although this NPC predictive model was tested on a 5‐year unbalanced real‐world data set, an extended cohort with a larger sample size and a longer follow‐up are necessary to evaluate its validity.

## 5 CONCLUSIONS

Individual EMRs represent an inexpensive and accessible data source. Our study used ML models built using such data, thus taking advantage of the opportunity to more reliably predict the occurrence of NPC. Applying our predictive model can help shorten the prediagnostic period in patients with NPC, and patients identified by the model as high‐risk should be promptly referred for confirmatory tests.

## AUTHOR CONTRIBUTIONS

**Jeng‐Wen Chen:** Conceptualization (equal); funding acquisition (equal); investigation (equal); methodology (equal); project administration (equal); resources (equal); visualization (equal); writing – original draft (equal); writing – review and editing (equal). **Shih‐Tsang Lin:** Conceptualization (equal); data curation (equal); funding acquisition (equal); project administration (equal); resources (equal). **Yi‐Chun Lin:** Conceptualization (equal); data curation (equal); formal analysis (equal); methodology (equal); software (equal); visualization (equal). **Bo‐Sian Wang:** Data curation (equal); methodology (equal). **Yu‐Ning Chien:** Conceptualization (equal); formal analysis (equal); investigation (equal); methodology (equal); project administration (equal); resources (supporting); validation (equal); visualization (equal); writing – original draft (equal); writing – review and editing (equal). **Hung‐Yi Chiou:** Conceptualization (equal); funding acquisition (equal); project administration (equal); supervision (equal); validation (equal).

## FUNDING INFORMATION

This study was supported by three resources: (1) The National Science and Technology Council of the Republic of China (Taiwan) grant (NSTC 110‐2511‐H‐567‐001‐MY2, NSTC 112‐2410‐H‐567‐001‐MY3, NSTC 112‐2314‐B‐845‐001) and (2) Cardinal Tien Hospital grant (CTH109A‐2218, CTH110A‐2204) and Cardinal Tien Junior College of Health Care and Management grant (CTCN‐109C‐09). The funders had no role in the design and conduct of the study; data collection, management, analysis, and interpretation; manuscript preparation, review, or approval; and the decision to submit the manuscript forpublication.

## CONFLICT OF INTEREST STATEMENT

None declared. All authors disclose that they have no relevant relationships.

## Supporting information


Appendix S1.

## Data Availability

Research data are not shared. Data sharing is not applicable to this article, because the NHIRD data is managed by the government and researchers can only analyze the data but cannot obtain it.
