# Supplementary material for: Early detection of nasopharyngeal carcinoma through machine‐learning‐driven prediction model in a population‐based healthcare record database
Source: Cancer Med. 2024 Mar 28;13(7):e7144. doi: 10.1002/cam4.7144 (PMC10973879; doi:10.1002/cam4.7144)
Supplement: Supplementary file 1 — Appendix S1. [file CAM4-13-e7144-s001.docx]

**Supplement**

Manuscript Title: Development and Validation of a Machine Learning Model for Nasopharyngeal Carcinoma Risk Assessment in a Population-Based Health-Care Records Database

**eTable 1.** Definition of features.

| **Features** | | **Description** | | **Level of Feature** |
| --- | --- | --- | --- | --- |
| **Category I. Participant Demographics** | | | | |
| Sex | | Male, female | | 2 |
| Age | | Age at index date | | Continuous |
| **Category II. Potential Pre-NPC Symptom-Related Diagnostic Codes** | | | | |
| **SN** | ***ICD-9-CM* code** | **Description** | | **Level of Feature** |
| 1 | 229 | Benign neoplasm of other and unspecified sites | | Indicator |
| 2 | 238.8 | Neoplasm of uncertain behavior of other specified sites | | Indicator |
| 3 | 238.9 | Neoplasm of uncertain behavior, site unspecified | | Indicator |
| 4 | 239.8 | Neoplasm of unspecified nature of other specified sites | | Indicator |
| 5 | 239.9 | Neoplasm of unspecified nature, site unspecified | | Indicator |
| 6 | 349.89 | Other specified disorders of nervous system | | Indicator |
| 7 | 349.9 | Unspecified disorders of nervous system | | Indicator |
| 8 | 350 | Trigeminal nerve disorders | | Indicator |
| 9 | 352 | Disorders of other cranial nerves | | Indicator |
| 10 | 368.2 | Diplopia | | Indicator |
| 11 | 368.3 | Other disorders of binocular vision | | Indicator |
| 12 | 381.1 | Chronic serous otitis media | | Indicator |
| 13 | 382 | Suppurative and unspecified otitis media | | Indicator |
| 14 | 383 | Mastoiditis and related conditions | | Indicator |
| 15 | 388 | Other disorders of ear | | Indicator |
| 16 | 389 | Hearing loss | | Indicator |
| 17 | 472 | Chronic pharyngitis and nasopharyngitis | | Indicator |
| 18 | 473 | Chronic sinusitis | | Indicator |
| 19 | 474 | Chronic disease of tonsils and adenoids | | Indicator |
| 20 | 478.1 | Other diseases of upper respiratory tract | | Indicator |
| 21 | 784.2 | Swelling, mass, or lump in head and neck | | Indicator |
| 22 | 784.7 | Epistaxis | | Indicator |
| 23 | 784.8 | Hemorrhage from throat | | Indicator |
| 24 | 784.9 | Other symptoms involving head and neck | | Indicator |
| 25 | 786.3 | Hemoptysis | | Indicator |
| 26 | 215.0 | Benign neoplasm of connective and other soft tissue of head, face, and neck | | Indicator |
| 27 | 378.5 | Paralytic strabismus | | Indicator |
| 28 | 784.0 | Headache | | Indicator |
| **Category III. Potential NHI Claim Codes of Pre-NPC Symptom-Related Procedures, Treatments, or Laboratory Tests** | | | | |
| **SN** | **NHI Code** | **Description** | | **Levels of Feature** |
| 1 | 14046B | EBV VCA IgG, IgM, IgA, IFA Method, Each | | Indicator |
| 2 | 12065B | EBV Capsid Ab | | Indicator |
| 3 | 14047B | EBNA Ab | | Indicator |
| 4 | 22003C | Impedance Audiometry | | Indicator |
| 5 | 22004C | Tympanometry | | Indicator |
| 6 | 22028C | Eustachian Tube Function Test | | Indicator |
| 7 | 28002C | Nasopharyngolaryngoscopy | | Indicator |
| 8 | 28003C | Sinoscopy | | Indicator |
| 9 | 28004C | Laryngoscopy | | Indicator |
| 10 | 29004C | Tympanic Aspiration | | Indicator |
| 11 | 29005C | Myringeal Puncture, Unilateral | | Indicator |
| 12 | 29006C | Middle Ear Cavity Puncture | | Indicator |
| 13 | 54005C | Eustachian Tube Inflation, Unilateral | | Indicator |
| 14 | 54006C | Eustachian Tube Inflation, Bilateral | | Indicator |
| 15 | 54010C | Simple Epistaxis, Anterior | | Indicator |
| 16 | 54011C | Complicated Epistaxis, Posterior | | Indicator |
| 17 | 54013C | Intranasal Cauterization | | Indicator |
| 18 | 54021C | Anterior Nasal Packing | | Indicator |
| 19 | 54022C | Posterior Nasal Packing | | Indicator |
| 20 | 54023C | Removal of Nasal Packing | | Indicator |
| 21 | 54036C | Tympanocentesis | | Indicator |
| 22 | 84007C | Myringotomy under Microscope or Telescope | | Indicator |
| 23 | 84015B | Myringotomy with Ventilation Tube Insertion under Microscope | | Indicator |
| 24 | 19012C | Head and Neck Soft Tissue Echo | | Indicator |
| **Category IV. Combined Feature of Diagnostic Codes (CFD)** | | | | |
| **SN** | **Acronym** | **Description** | ***ICD-9-CM* Codes** | **Levels of Feature** |
| 1 | D_COMP3_OP_D1 | Head and Neck Mass | 229, 238.8, 238.9, 239.8, 239.9, 784.2, 215.0 | Indicator |
| 2 | D_COMP3_OP_D2 | Nasal Symptoms | 472, 473, 474, 478.1 | Indicator |
| 3 | D_COMP3_OP_D3 | Aural Symptoms | 381.1, 382, 383, 388, 389 | Indicator |
| 4 | D_COMP3_OP_D4 | Headache | 784.0 | Indicator |
| 5 | D_COMP3_OP_D5 | Bleeding | 784.7, 784.8, 786.3 | Indicator |
| 6 | D_COMP3_OP_D6 | Neurological Sign | 349.89, 349.9, 350, 352, 368.2, 368.3, 378.5 | Indicator |
| 7 | D_COMP3_OP_D7 | Others | 784.9, 075 | Indicator |
| **Category V. Combined Features of Procedures, Treatments, and Laboratory Tests (CFPTLT)** | | | | |
| **SN** | **Acronym** | **Description** | ***ICD-9-CM* Codes** | **Levels of Feature** |
| 1 | D_COMP3_TRT1 | Head and Neck Mass Management | 19012C | Indicator |
| 2 | D_COMP3_TRT2 | Nasal Symptoms Management | 28002C, 28003C, 28004C | Indicator |
| 3 | D_COMP3_TRT3 | Aural Symptoms Management | 22003C, 22004C, 22028C, 29004C, 29005C, 29006C, 54005C, 54006C, 54036C, 84007C, 84015B | Indicator |
| 4 | D_COMP3_TRT4 | Serum Markers | 14046B, 12065B, 14047B | Indicator |
| 5 | D_COMP3_TRT5 | Bleeding Management | 54010C, 54011C, 54013C, 54021C, 54022C, 54023C | Indicator |

**eTable 2. Feature selection combination used to train machine learning algorithms**

| **Acronym** | **Features used in feature selection model** | **No. of Features** |
| --- | --- | --- |
| Fea_comb1 | Category I (Participant Demographics) + Category IV (Combined Features of Diagnostic Codes, CDF) | 2 + 7  (total 9) |
| Fea_comb2 | Category I (Participant Demographics) + Category V (Combined Features of Procedures, Treatments, and Laboratory Tests, CFPTLT) | 2 + 5  (total 7) |
| Fea_comb3 | Category I (Participant Demographics) + Category IV (Combined Features of Diagnostic Codes, CDF) + Category V (Combined Features of Procedures, Treatments, and Laboratory Tests, CFPTLT) | 2 + 7 + 5  (total 14) |
| Fea_comb4 | Category I (Participant Demographics) + Category II (Potential Pre-NPC Symptom-Related Diagnostic Codes) | 2 + 28  (total 30) |
| Fea_comb5 | Category I (Participant Demographics) + Category III (Potential NHI Claim Codes of Pre-NPC Symptom-Related Procedures, Treatments, or Laboratory Tests) | 2 + 24  (total 26) |
| Fea_comb6 | Category I (Participant Demographics) + Category II (Potential Pre-NPC Symptom-Related Diagnostic Codes) + Category III (Potential NHI Claim Codes of Pre-NPC Symptom-Related Procedures, Treatments, or Laboratory Tests) + Category IV (Combined Features of Diagnostic Codes, CDF) + Category V (Combined Features of Procedures, Treatments, and Laboratory Tests, CFPTLT) | 2 + 28 + 24 + 7 + 5  (total 66) |

**eTable 3: Machine learning algorithms’ acronym, name, and brief description**

| **Acronym** | **Name** |
| --- | --- |
| MARS | Multivariate Adaptive Regression Splines |
| LGB | Light Gradient Boosting Machine |
| XGB | eXtreme Gradient Boosting |
| RF | Random Forest |
| LG | Logistics Regression |

| 1. **Predictive modeling using data from 90 days before the index date** | 1. **Predictive modeling using data from 120 days before the index date** |
| --- | --- |
| **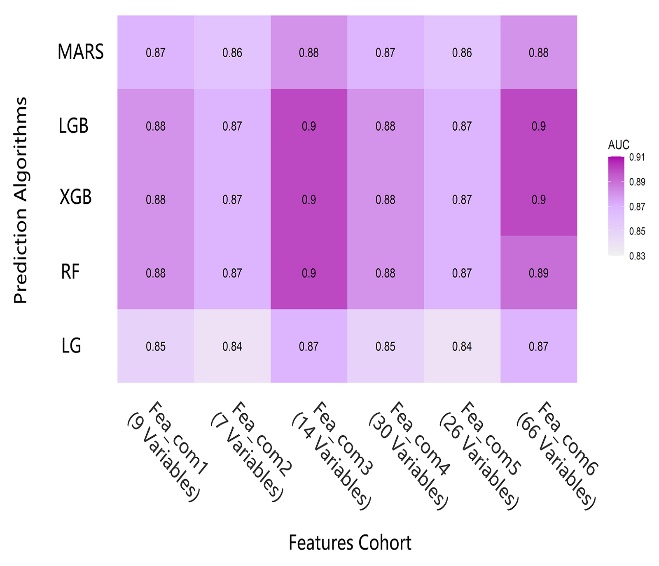** | **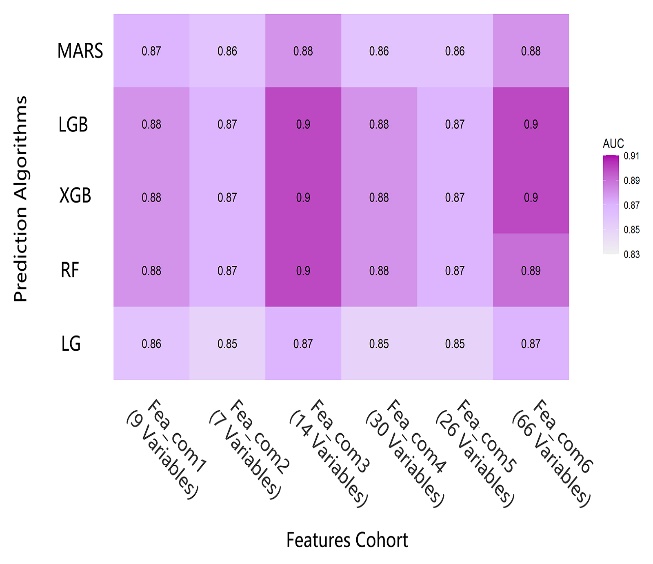** |
| 1. **Predictive modeling using data from 150 days before the index date** | 1. **Predictive modeling using data from 180 days before the index date** |
| **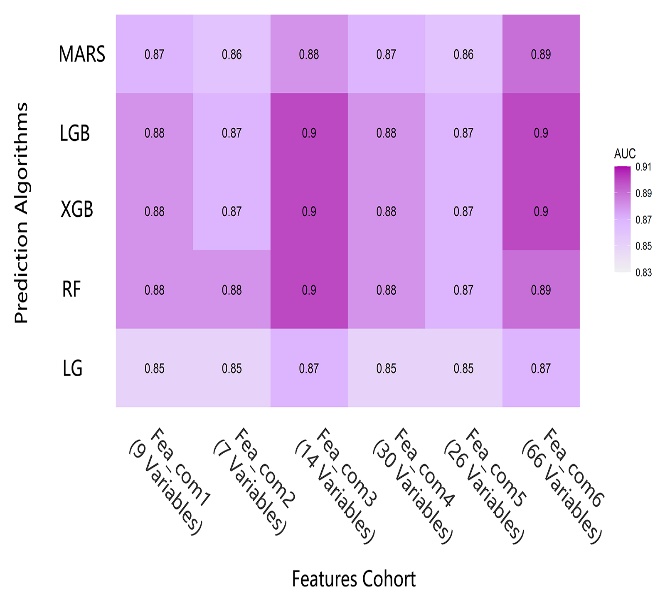** | **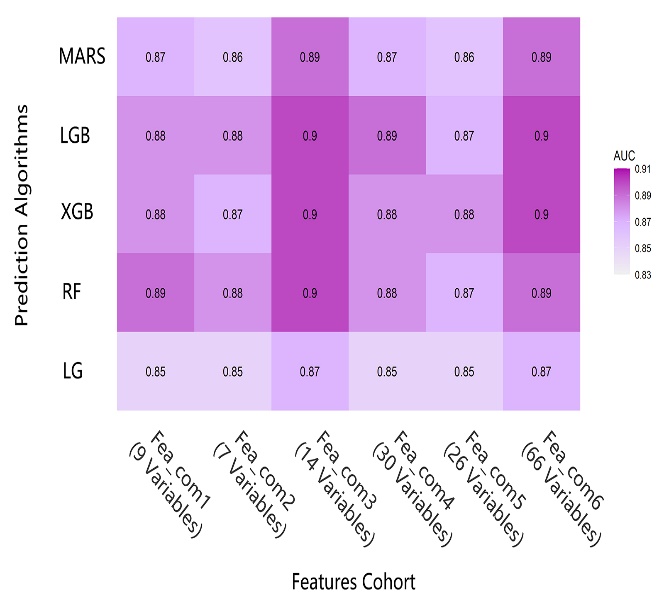** |
| 1. **Predictive modeling using data from 360 days before the index date** |  |
| **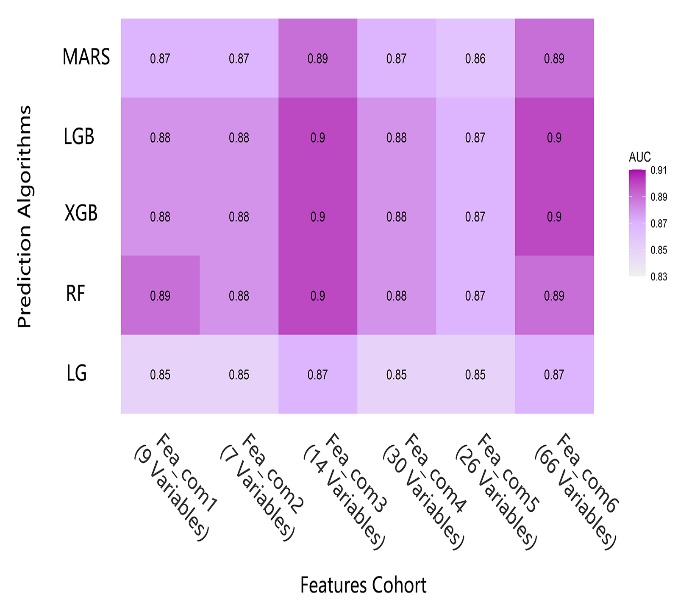** |  |

**eFigure 1.** Heatmap depicting model performance prediction (AUROC) of feature selection and machine learning algorithms using data from 90, 120,150, 180, and 360 days before the index date

**eTable 4. AUROC value in training set of machine learning algorithms with Fea_Comb3 Model (collected medical claims from 90, 120, 150, 180, and 360 days before index date)**

|  | **Days before diagnosis** | | | | |
| --- | --- | --- | --- | --- | --- |
|  | **90 days** | **120 days** | **150 days** | **180 days** | 1. **days** |
| MARS | 0.88 | 0.88 | 0.88 | 0.89 | 0.88 |
| LGB | 0.90 | 0.90 | 0.90 | 0.90 | 0.90 |
| XGB | 0.90 | 0.90 | 0.90 | 0.90 | 0.90 |
| RF | 0.90 | 0.90 | 0.90 | 0.90 | 0.91 |
| LG | 0.88 | 0.88 | 0.87 | 0.87 | 0.87 |
| **Mean** | **0.89** | **0.89** | **0.89** | **0.89** | **0.89** |

**eTable 5. Performance metrics of machine learning algorithms based on selected feature of Fea_Comb3 model and 360 days before index date**

|  | **Days before diagnosis** | | | |
| --- | --- | --- | --- | --- |
|  | **Sensitivity** | **Specificity** | **Balanced Accuracy** | **AUROC** |
| **A. AUROC value in the training set** | | | | |
| MARS | 0.79 | 0.79 | 0.79 | 0.88 |
| LGB | 0.82 | 0.78 | 0.80 | 0.90 |
| XGB | 0.80 | 0.80 | 0.80 | 0.90 |
| RF | 0.81 | 0.81 | 0.81 | 0.91 |
| LG | 0.78 | 0.77 | 0.78 | 0.87 |
| **B. AUROC value in the validation set** | | | | |
| MARS | 0.78 | 0.79 | 0.79 | 0.88 |
| LGB | 0.81 | 0.78 | 0.80 | 0.89 |
| XGB | 0.81 | 0.80 | 0.80 | 0.89 |
| RF | 0.80 | 0.79 | 0.80 | 0.90 |
| LG | 0.77 | 0.77 | 0.77 | 0.87 |
| **C. AUROC value in the test set** | | | | |
| MARS | 0.64 | 0.80 | 0.72 | 0.82 |
| LGB | 0.65 | 0.79 | **0.72** | **0.82** |
| XGB | 0.64 | 0.81 | 0.72 | 0.82 |
| RF | 0.64 | 0.81 | 0.73 | 0.82 |
| LG | 0.62 | 0.79 | 0.71 | 0.80 |

**eTable 6. Descriptive statistics of high-risk and low-risk groups defined in real world data using risk prediction model.**

| **Characteristic** | **Cohort, No. (%)** | | ***P* value** |
| --- | --- | --- | --- |
|  | **Low Risk Group**  **(n = 731175)** | **High Risk Group**  **(n = 244457)** |  |
| **Age, mean (SD), y** | 31.36 (20.22) | 52.12 (11.46) | <.0001 |
| **Sex** |  |  | <.0001 |
| Male | 256217(35.04%) | 223447(91.41%) |  |
| Female | 474958(64.97%) | 21010(8.59%) |  |
| **Potential Pre-NPC Symptom-Related Diagnostic Codes** |  |  |  |
| Benign neoplasm of other and unspecified sites | 15(0.00%) | 338(0.14%) | <.0001 |
| Neoplasm of uncertain behavior of other specified sites | 0(0.00%) | 6(0.00%) | <.0001 |
| Neoplasm of uncertain behavior, site unspecified | 0(0.00%) | 44(0.02%) | <.0001 |
| Neoplasm of unspecified nature of other specified sites | 3(0.00%) | 80(0.03%) | <.0001 |
| Neoplasm of unspecified nature, site unspecified | 4(0.00%) | 56(0.02%) | <.0001 |
| Other specified disorders of nervous system | 37(0.00%) | 216(0.09%) | <.0001 |
| Unspecified disorders of nervous system | 199(0.03%) | 788(0.32%) | <.0001 |
| Trigeminal nerve disorders | 34(0.01%) | 366(0.15%) | <.0001 |
| Disorders of other cranial nerves | 5(0.00%) | 78(0.03%) | <.0001 |
| Diplopia | 2(0.00%) | 30(0.01%) | <.0001 |
| Other disorders of binocular vision | 5(0.00%) | 24(0.01%) | <.0001 |
| Chronic serous otitis media | 140(0.02%) | 140(0.06%) | <.0001 |
| Suppurative and unspecified otitis media | 3055(0.42%) | 2441(1.00%) | <.0001 |
| Mastoiditis and related conditions | 8(0.0011%) | 26(0.01%) | <.0001 |
| Other disorders of ear | 398(0.05%) | 4532(1.85%) | <.0001 |
| Hearing loss | 270(0.04%) | 1664(0.68%) | <.0001 |
| Chronic pharyngitis and nasopharyngitis | 9428(1.29%) | 11 386(4.66%) | <.0001 |
| Chronic sinusitis | 693(0.09%) | 1706(0.70%) | <.0001 |
| Chronic disease of tonsils and adenoids | 348(0.05%) | 545(0.22%) | <.0001 |
| Other diseases of upper respiratory tract | 3941(0.54%) | 3129(1.28%) | <.0001 |
| Swelling, mass, or lump in head and neck | 32(0.00%) | 390(0.16%) | <.0001 |
| Epistaxis | 1496(0.20%) | 1207(0.49%) | <.0001 |
| Hemorrhage from throat | 2(0.00%) | 8(0.00%) | <.0001 |
| Other symptoms involving head and neck | 75(0.01%) | 32(0.01%) | .2469 |
| Hemoptysis | 16(0.00%) | 362(0.15%) | <.0001 |
| Benign neoplasm of connective and other soft tissue of head, face, and neck | 14(0.00%) | 235(0.10%) | <.0001 |
| Paralytic strabismus | 11(0.00%) | 51(0.02%) | <.0001 |
| Headache | 19 357(2.65%) | 15 086(6.17%) | <.0001 |
| **Potential NHI Claim Codes of Pre-NPC Symptom-Related Procedures, Treatments, or Laboratory Tests** |  |  |  |
| EBV VCA IgG, IgM, IgA, IFA Method, Each | 79(0.01%) | 150(0.06%) | <.0001 |
| EBV Capsid Ab | 1(0.00%) | 0(0.00%) | .5631 |
| EBNA Ab | 36(0.00%) | 63(0.03%) | <.0001 |
| Impedance Audiometry | 104(0.01%) | 719(0.29%) | <.0001 |
| Tympanometry | 610(0.08%) | 1638(0.67%) | <.0001 |
| Eustachian Tube Function Test | 0(0.00%) | 40(0.02%) | <.0001 |
| Nasopharyngolaryngoscopy | 36(0.01%) | 1734(0.71%) | <.0001 |
| Sinoscopy | 2(0.00%) | 395(0.16%) | <.0001 |
| Laryngoscopy | 7(0.00%) | 861(0.35%) | <.0001 |
| Tympanic Aspiration | 3(0.00%) | 31(0.01%) | <.0001 |
| Myringeal Puncture, Unilateral | 1(0.00%) | 2(0.00%) | .0963 |
| Middle Ear Cavity Puncture | 0(0.00%) | 6(0.00%) | <.0001 |
| Eustachian Tube Inflation, Unilateral | 14(0.00%) | 144(0.06%) | <.0001 |
| Eustachian Tube Inflation, Bilateral | 17(0.00%) | 165(0.07%) | <.0001 |
| Simple Epistaxis, Anterior | 914(0.13%) | 737(0.30%) | <.0001 |
| Complicated Epistaxis, Posterior | 4(0.00%) | 17(0.01%) | <.0001 |
| Intranasal Cauterization | 1(0.00%) | 12(0.00%) | <.0001 |
| Anterior Nasal Packing | 6(0.00%) | 13(0.00%) | <.0001 |
| Posterior Nasal Packing | 3(0.00%) | 4(0.00%) | .0501 |
| Removal of Nasal Packing | 23(0.00%) | 127(0.05%) | <.0001 |
| Tympanocentesis | 5(0.00%) | 66(0.03%) | <.0001 |
| Myringotomy under Microscope or Telescope | 2(0.00%) | 27(0.01%) | <.0001 |
| Myringotomy with Ventilation Tube Insertion under Microscope | 34(0.00%) | 45(0.02%) | <.0001 |
| Head and Neck Soft Tissue Echo | 313(0.04%) | 1313(0.54%) | <.0001 |
